# A single heterochronic blood exchange reveals rapid inhibition of multiple tissues by old blood

**DOI:** 10.1038/ncomms13363

**Published:** 2016-11-22

**Authors:** Justin Rebo, Melod Mehdipour, Ranveer Gathwala, Keith Causey, Yan Liu, Michael J. Conboy, Irina M. Conboy

**Affiliations:** 1 Department of Bioengineering and QB3 Institute, 174 Stanley Hall, University of California, Berkeley, Berkeley, California 94720-3220, USA; 2 SENS Research Foundation, 110 Pioneer Way, Suite J, Mountain View, California 94041, USA

## Abstract

Heterochronic parabiosis rejuvenates the performance of old tissue stem cells at some expense to the young, but whether this is through shared circulation or shared organs is unclear. Here we show that heterochronic blood exchange between young and old mice without sharing other organs, affects tissues within a few days, and leads to different outcomes than heterochronic parabiosis. Investigating muscle, liver and brain hippocampus, in the presence or absence of muscle injury, we find that, in many cases, the inhibitory effects of old blood are more pronounced than the benefits of young, and that peripheral tissue injury compounds the negative effects. We also explore mechanistic explanations, including the role of B2M and TGF-beta. We conclude that, compared with heterochronic parabiosis, heterochronic blood exchange in small animals is less invasive and enables better-controlled studies with more immediate translation to therapies for humans.

## Abstract (web summary)

Joining the circulatory system of an old with a young animal has been shown to rejuvenate old tissues. Here the authors describe a comparatively simple blood infusion system that allows for the controlled exchange of blood between two animals, and study the effects of a single exchange on various tissues.

Technological developments and progress in our understanding of disease have eliminated many previously common sources of mortality. Along with our age shifting demographic, however, a host of new debilitating age-associated disorders have emerged. The medical costs of managing an aging world poses significant economic and social challenges and will ultimately require a long-term solution. One way to increase healthy longevity would be to rejuvenate the regenerative and repair capacity of aged tissues. Published work suggests restoring the circulatory environment of aged tissues back to a productive, young, composition may help to rapidly and broadly enhance the maintenance and repair of multiple old organs, combat degeneration and extend health span^1^,^2^,^3^,^4^,^5^,^6^,^7^,^8^.

The effects seen by heterochronic parabiosis, the surgical joining of two animals of different ages, include rejuvenation of multiple tissues in the old partner, and are often simplistically assumed to be caused by the exchange of macromolecules found in plasma, however, parabiosis is far more complex. For example, old animals with young partners have, through the shared circulation, continuous access to the young organs, which regulate metabolic homeostasis, wound clearance and inflammation, and provide blood oxygenation to the animals^3^,^5^. Old mice attached to young animals also benefit from environmental enrichment and youthful pheromones, which may play a role in neuronal plasticity and neurogenesis^9^,^10^. The young parabiont partially maintains an additional aged body with deteriorating organs, chronic inflammation and skewed immune responses. Additionally, young and old organ systems have an opportunity to ‘adapt' to prolonged sharing of circulatory milieus and thus change their local influences on resident stem cells. All of the above could contribute to the observed differences in regenerative responses.

One conclusion from recent studies on heterochronic parabiosis is that the regenerative capacity of old tissue stem cells in all three germ layer derivatives can be enhanced by the young systemic milieu^3^,^4^,^11^,^12^,^13^. It is tempting to assume that young plasma has pro-regenerative factors, and indeed administration of young plasma to aged mice improved their cognition^14^. However, the effects of young blood plasma on stem cells in brain or other tissues have not been studied, and it remains to be discovered whether and which plasma factors would be active enough to influence neurogenesis or cognition at small doses when added to an aged circulation, and would be able to cross the blood–brain barrier to have positive or negative central effects. Thus far, only heterochronic parabiosis has been shown to enhance myogenesis, hepatogenesis, bone regrowth, neurogenesis, cognition and the numbers of dendritic spines in old mice. Most importantly, the positive effects of heterochronic parabiosis are robust for muscle, lesser for liver and marginal for neurogenesis; and a significant inhibition of even young tissue stem cells by the aged circulatory milieu takes place^2^,^4^,^5^,^8^,^11^,^15^.

In contrast to the permanent anastomosis of parabiosis, we developed a blood exchange system where animals are connected and disconnected at will, removing the influence of shared organs, adaptation to being joined and so on. The effects of heterochronic blood exchange were examined with respect to all three germ layer derivatives: injured-regenerating muscle, ongoing liver cell proliferation and adiposity, and in the brain, hippocampal neurogenesis, and this time in the presence versus absence of muscle injury. Most surprisingly, the onset of the influence of heterochronic blood exchange on myogenesis, neurogenesis and hepatogenesis turned out to be within a few days. Notably, the outcome of heterochronic blood exchange is also different from heterochronic parabiosis, particularly for neurogenesis where our results suggest that old blood is far more inhibitory to tissue health than that young blood is rejuvenative, and that peripheral tissue injury compounds the negative effects of old blood on young neurogenesis. Heterochronic blood exchange enhances old muscle repair without inhibition of young, and old hepatogenesis is improved and fibrosis and adiposity are decreased, while young hepatogenesis becomes diminished. Moreover, our studies demonstrate a rapid increase in beta-2 microglobulin (B2M) in young tissues by old blood; and this phenotype is not from elevated circulating B2M in old mice (as there is none), suggesting that another age-specific systemic molecule raises B2M in the young organs. Blood exchange in small animals enables well-controlled studies with more rapid translation for therapy for humans.

## Results

### Blood exchange between mice

To develop a better-controlled experimental system where only blood is exchanged, we developed a small animal blood exchange device, which operates similarly to a previously published circulatory cell-scrubbing device^16^. It consists of a computer controlled microfluidic peristaltic pump circuit and computer controlled extracorporeal blood manipulation system (Supplementary Fig. 1 and Supplementary Movie 1). A major design constraint of small animal blood manipulation that we have addressed is the low volume of total blood that can be removed from a small animal at once. It is not prudent to remove more than 10% of an animal's blood at once, and mice contain 5–8% (w/w) blood. This translates to ∼150 μl of blood that can be removed from a 30 g mouse. Small volume microfluidic blood manipulation systems exist for lab on a chip and other diagnostic applications^17^, however, our device is the first to allow for continuous blood flow as required for larger scale experimental applications in live mice (Supplementary Movie 1). In contrast to parabiosis, where joint circulation is established in ∼7–10 days through growth of skin capillaries, blood exchange is instantaneous and well controlled by the device. The procedure is less invasive than parabiosis as it does not involve as much invasive surgery, only the catheterization of a jugular vein (Supplementary Fig. 1). The exchanged blood is visualized in the tubing and the exchange volumes are easily measured.

Using this small animal device we have exchanged blood between 4 pairs of young to old mice, using 4 pairs of isochronic, young to young exchanges and 4 pairs of isochronic, old to old exchanges, as controls. Virtually 100% animal viability is maintained when two series of 15 exchanges of 150 μl of blood per series are performed over the course of 24 h, establishing a blood equilibrium similar to parabiosis between the pairs in a fraction of the time. This regiment was employed for the studies and will be referred to as a single procedure of blood exchange thereafter.

### Young blood improves old muscle regeneration

**Figure 1:**
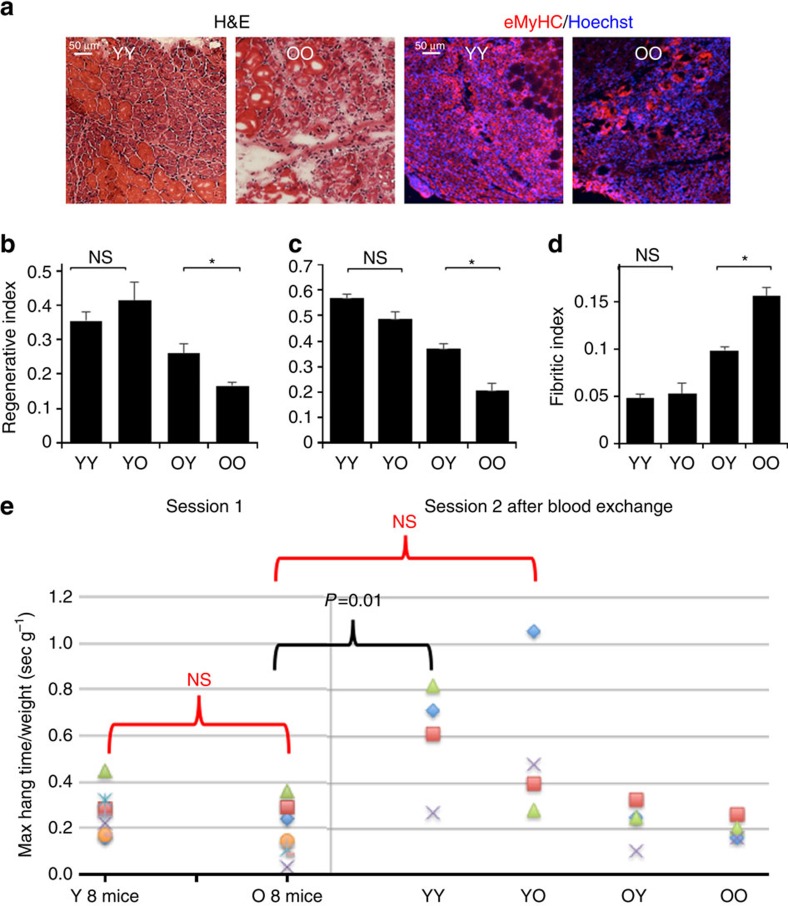
Heterochronic blood exchange effects on muscle regeneration and performance. One day after blood exchange mice were injured by intramuscular injections of CTX into TA. Five days after injury, TA muscles were isolated, cryo-sectioned and analysed. (**a**) TA muscles from young mice receiving young blood (YY), young mice receiving old blood (YO), old mice receiving young blood (OY) and old mice receiving old blood (OO) were analysed by haematoxylin and eosin (H&E) staining and immunofluorescence with anti-eMyHC antibody. Representative images show an injury site and nascent de-novo formed eMyHC+ myofibers which are smaller in size with central nuclei than uninjured myofibers. Scale bar, 50 μm for H&E panel and 25 μm for immunofluorescence panel. (**b**,**c**) Regeneration indices ±s.e.m. were quantified from H&E images (**b**) and eMyHC images (**c**) by counting the number of nascent de-novo formed myofibers and dividing by the total number of nuclei present at the injury/regeneration site. By H&E: *P<0.05 *N*=4 per group. Significant students *t* test differences exist between YO and OY (*P*=0.045), YY and OY (*P*=0.043), YY and OO (*P*=0.0004), YO and OO (*P*=0.0042) and between OY and OO (*P*=0.015). By eMyHC: *P<0.05, *N*=4 per group; OY and OO *P*=0.041, YY and OO *P*=0.00009, and YO to OO *P*=0.001. (**d**) Fibrotic/inflammatory indexes were quantified as total injury area minus regenerated myofiber area, per injury site, using the H&E images^15^. *T*-test **P<0.005, *n*=3–4 per group. Muscle from old to old isochronic exchange had diminished regenerative capacity and more fibrosis, as compared with muscle from young to young isochronic exchange. Heterochronic blood exchange significantly improved regeneration of old muscle after experimental injury and reduced fibrosis, but no significant decline in young muscle regeneration was seen. (**e**) A four-limb hanging test was conducted with isochronically and heterochronically transfused mice that were not injured, before and at 6 days after the blood exchange. Maximal hanging time was multiplied by body weight (hang index). *T*-test *n*=4–8, *P*=0.01 YY post transfusion compared with O training, and YO, OY, OO post-transfusion performance. Y to O training and YO, OY and OO were NS=not statistically different.

One day after the blood exchange, tibiallis anterior (TA) hind leg muscles of all mice were injured by cardiotoxin (CTX) and 5 days later this muscle, as well as non-injured livers and brains were isolated postmortem. The efficiency of muscle regeneration was determined in a manner identical to the heterochronic parabiosis studies^3^,^4^. TA muscles were injected with CTX 1 (CTX, Sigma, 0.1 mg ml^−1^). Ten micrometre muscle cryo-sections were prepared from TA muscle, which was isolated at 5 days post CTX injury. These cryo-sections were analysed by haematoxylin and eosin (H&E), staining and by eMyHC immuno-detection followed by microscopy and quantification of the percent of *de- novo* small eMyHC+ myofibers with centrally located nuclei that robustly appear in young, but are less in the old injured muscle, which typically shows more inflammation and incipient fibrosis. As shown in Fig. 1a–c, a single procedure of heterochronic blood exchange significantly improved the regeneration of old muscle after experimental injury (both when assayed by H&E staining or eMyHC immunofluorescence), while there was no statistically significant decline in the robust regeneration of the young muscle. The numbers of de-novo myofibers were slightly higher for all cohorts when counted by the more robust eMyHC immunofluorescence method as compared with H&E, but the relative differences between the YY, YO, OY and OO cohorts remained virtually the same. We also examined de-novo fibre size (minimal Feret diameter, as published^6^,^18^), as expected the regenerating fibre size declined with age but interestingly was increased in old mice transfused with young blood, while remaining unchanged in young mice transfused with old blood (Supplementary Fig. 2). Importantly, the degree of fibrosis (a culprit of muscle aging) was also reduced by the young blood exchange and was not increased in the young mice transfused with old blood (Fig. 1d).

### Old blood inhibits young performance

To assay functional performance, the blood exchange studies were repeated without muscle injury, and a four-limb hanging test was applied to the isochronic and heterochronic cohorts 6 days after the blood exchange (for example, the same time frame when muscle repair was studied in the injured mice). In this test animal strength, endurance and learning are all measured: mice hang inverted from a 1 cm mesh screen over soft bedding, and the time until the mouse drops is recorded over three trials, and the maximal time multiplied by the weight is expressed as hanging index^19^. Interestingly, exchange with old blood markedly diminished the maximal hanging index of young animals (3 out of 4 mice) but there was no increase in this parameter for the old mice transfused with young blood (Fig. 1e). Of note, the initial hanging indices in training session were not significantly different between the young and old mice, but young animals transfused with young blood became statistically better than the old mice after the training session, while young mice transfused with old blood remained statistically undistinguishable from the old cohorts (Fig. 1e).

These data extrapolate the findings obtained with heterochronic parabiosis^4^, and establish that the beneficial effects of young blood for the regeneration of old muscle take place right away and without the contribution of young organ systems or altered activity levels between the isochronic and heterochronic pairs. Moreover, while one exchange of young blood improves muscle regeneration in old animals, it does not improve the functional performance as measured by the hanging test, while in young animals the functional performance declines very rapidly after one exchange of old blood.

### Hippocampal neurogenesis responds to old blood and injury

**Figure 2:**
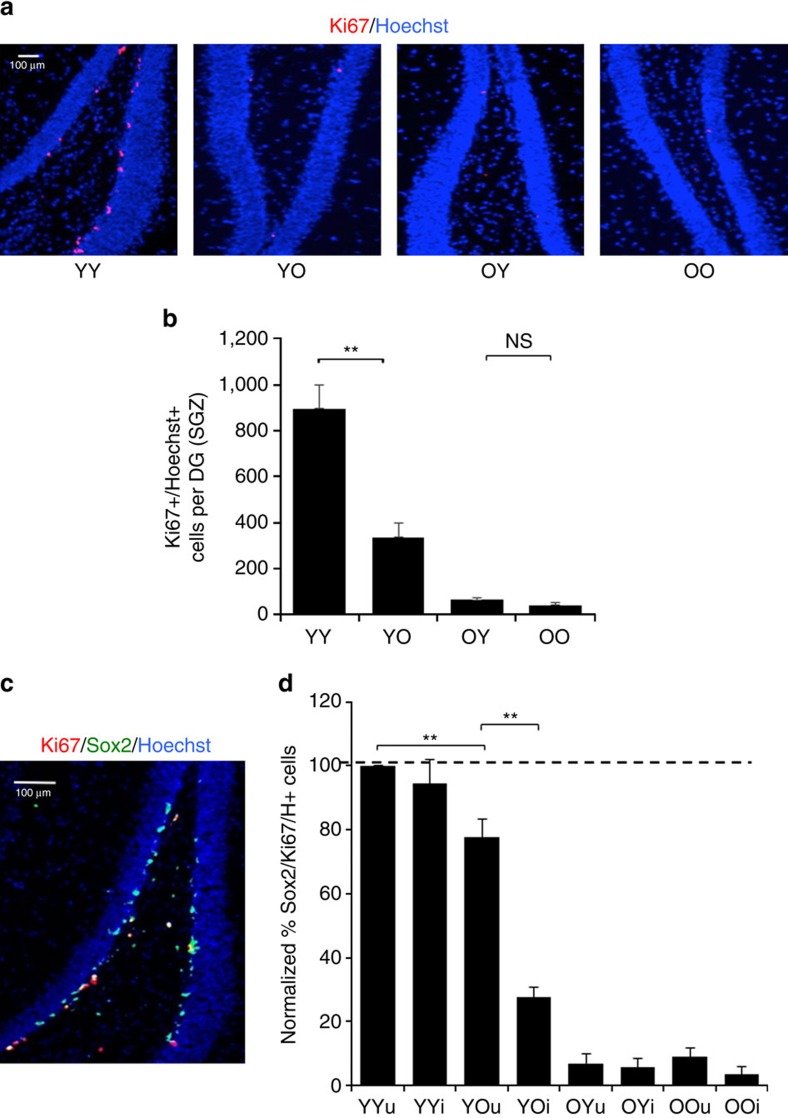
Heterochronic blood exchange reduces the proliferative potential of old neural stem cells. The effects of isochronic and heterochronic blood exchange on SGZ neurogenesis were determined in animals from Fig. 1, with and without muscle injury. (**a**) Brains from YY, YO, OY and OO mice that had muscle injury were frozen and sectioned at 25 μm. Cryo-sections were immunostained for the proliferation marker Ki67 (red) and counterstained for nuclei (Hoechst, blue). Shown are representative images of the dentate gyrus (DG). Scale bar, 100 μm. (**b**) Proliferating (Ki67+/Hoechst+) cells in SGZ were quantified in serial 25-μm cryo-sections for each experimental cohort spanning the DG. Ki67+/Hoechst+ cells were clearly identifiable as seen in the enlarged inset image from **a**, outlined in white. Ki67+ SGZ cells decrease with age and also a decrease is seen in heterochronic young brains compared with the isochronic young controls. At the same time, there in no enhancement of SGZ cell proliferation occurring in heterochronic old brains as compared with the isochronic old controls. *T*-test **P<0.005. *N*=4, YY to YO (*P*=0.0034), OY (*P*=0.0002) and OO (*P*=0.000159), YO to OY (*P*=0.0047), and OO (*P*=0.0032). (**c**) Ki67 largely colocalized with Sox2 by immunodetection in of brains from YY, YO, OY and OO mice with and without the experimental muscle injury. Hoechst (blue) was used to label all nuclei. Representative image of YY cohort with muscle injury is shown. Scale bar, 100 μm. (**d**) Quantification of Ki67+/Sox2+/Hoechst+ cells per SGZ was performed for all blood exchange cohorts above; shown are the relative numbers compared with the in YY cohort without injury that is set to 100%. Similarly to SGZ Ki67+/Hoechst+ cells, the numbers of SGZ proliferating Sox2+ cells diminished with age and significantly decreased after exposure of young cells to old blood by a single procedure of exchange. Notably, neurogenesis was significantly attenuated in YO mice with muscle injury as compared with the uninjured animals of the same cohort (*P*=0.001). No significant positive effects on old Ki67+/Sox2+/Hoechst+ cells were detected with or without muscle injury. *n*=4, *P<0.05, **P<0.005.

The efficiency of hippocampal neurogenesis was determined similarly to refs 8 and (ref. 3). Mouse brains were collected and sectioned at 25 μm using a cryostat. Sections were fixed in 4% paraformaldehyde and immunostained with antibodies to Ki67, using Hoechst co-stain to detect all nuclei. The numbers of Ki67+ proliferating subgranular zone (SGZ) cells were quantified throughout the entire dentate gyrus (DG) of the hippocampus, as in ref. 8, where based on co-immunodetection of Sox-2, these Ki67+ SGZ cells are virtually all-neural stem cells. As shown in Fig. 2 and Supplementary Fig. 3, based on either SGZ Ki67+ or SGZ Ki67+/Sox2+ cell numbers, one exchange of heterochronic blood severely decreased hippocampal neurogenesis in young mice, and surprisingly, there was no significant positive effect in the old mice that had been exchanged with young blood. Of note, these were the same old animals that showed improvement in muscle regeneration and hepatogenesis (see below).

In the some of published heterochronic parabiosis work, muscle was injured in animals that were assayed for hippocampal neurogenesis^3^,^4^ and similar effects on neurogenesis were later seen in the absence of muscle injury^11^. We compared the influences of heterochronic blood exchange on neurogenesis in the presence and absence of muscle injury, to assay for potential changes in the brain that might be caused by additional stress and peripheral inflammation. While a statistically significant inhibition of young neurogenesis by old blood persisted, its magnitude was less in the absence of muscle injury (Fig. 2c). There was no enhancement of old neurogenesis by the young blood, with or without muscle injury (Fig. 2 and Supplementary Fig. 3). These data confirm the negative effects of the old systemic milieu on neurogenesis in young hippocampi and identify that such inhibition is very rapid and is uncoupled from influences of old organ systems, pheromones and changes in the environmental stimulation or exercise. Muscle injury after blood exchange might add to the magnitude of the negative effects of old blood on young neurogenesis, and even without muscle injury, young hippocampal neurogenesis quickly declines after one old blood exchange.

### Liver responds to heterochronic blood and muscle injury

**Figure 3:**
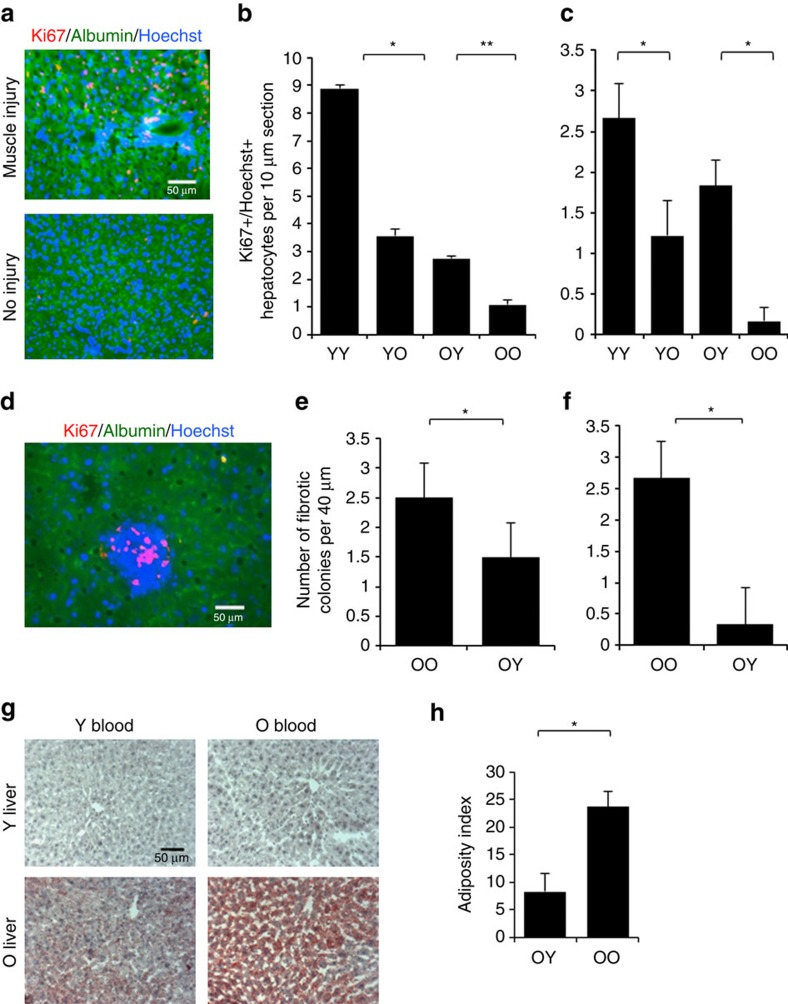
Heterochronic blood exchange effects on hepatogenesis and liver fibrosis and adiposity. (**a**) Livers from YY, YO, OY and OO mice with and without experimental muscle injury as above were cryo-sectioned at 10 μm and immuno-stained for Ki67 (red), hepatocyte marker albumin (green) and Hoechst (blue). Representative images show YY livers with and without injury. Scale bar, 50 μm. (B&C. Quantification of hepatocyte proliferation was by counting the average number of Ki67+,abumin+,Hoechst+ cells per 10 μm section from multiple sections of each blood exchange cohort. (**b**) Old hepatocyte showed increased proliferation and young hepatocytes showed less proliferation with heterochronic blood as compared with isochronic blood exchanges in animals with injured muscle (*t* test *P*=0.00028). (**c**) This trend continues without muscle injury, but the total numbers of proliferating hepatocytes decline by twofold, (*P*=0.02411). *P<0.05; **P<0.005; *n*=3–5. (**d**) As previously published^4^, there were fibrotic clusters exclusively in the old livers of small Ki67+ve, albumin negative Ki67+ cells. Scale bar, 50 μm, × 40 magnification. (**e**,**f**) Fibrotic index was calculated as the average number of albumin negative proliferative cell clusters per four 10 μm sections. The fibrotic index diminished in old mice exchanged with young blood with muscle injury (**e**) (*t* test *P*=0.048 *N*=4, *P<0.05) or without (**f**) (*t* test *P*=0.00776. *N*=3; **P<0.005). (**g**) Liver adiposity was assayed by Oil Red in 10 μm cryosections. Shown are representative images acquired at × 20 magnification. (**h**). Liver adiposity (red) was quantified by Image J, dramatically increased with age and was attenuated by young blood in old mice (*t* test *N*=3, *P*=0.022), while adiposity remained unchanged in young mice that were transfused with the old blood (see Supplementary Figure 4). Shown are means±s.e.m. for all histograms.

We next assayed the efficiency of ongoing hepatogenesis in mice that were and were not experimentally injured in their muscle, as in ref. 4 by co-immunodetection of a proliferation marker, Ki67 and a hepatocyte marker albumin, in 10 μm liver cryosections. As shown in Fig. 3a,b, the numbers of proliferating old hepatocytes were increased after a single procedure of heterochronic blood exchange, while the numbers of Ki67+albumin+ young hepatocytes declined on transfusion of old blood. The ongoing hepatogenesis in animals that did not experience muscle injury was much less prominent even in young mice, suggesting that hepatogenesis increases during muscle repair, but still the heterochronic effects of a single blood exchange were observed (Fig. 3a,b).

As previously reported^4^ there were many fibrotic areas in old livers, which at times had proliferating clusters of small albumin negative cells (Fig. 3c,d). Such areas were not present in young livers, and very interestingly the numbers of these fibrotic proliferative clusters declined in the livers of old animals that were exchanged with young blood, regardless of whether muscle was or was not injured (Fig. 3c,d).

As another metric for improvement in liver health we assayed liver tissue adiposity by Oil Red staining on 10 μm cryosections from the above-described animals. Old livers were markedly more positive for Oil Red, as compared with young and interestingly, transfusion with young blood somewhat reduced old liver adiposity, while there was no significant increase in young liver adiposity (Fig. 3e,f and Supplementary Fig. 4). These results demonstrate that heterochronic blood exchange and heterochronic parabiosis yield similar enhancement of old hepatogenesis and decline of young hepatogenesis; and suggest that muscle damage enhances ongoing hepatogenesis in young mice. Additionally, the fibrotic regions and adiposity rapidly decline in old livers after the exposure to young blood. Such effects manifest after just a single procedure of blood exchange and in the absence of the influences from heterochronic organ systems.

### B2M and TGF-beta as mechanisms are tissue specific

**Figure 4:**
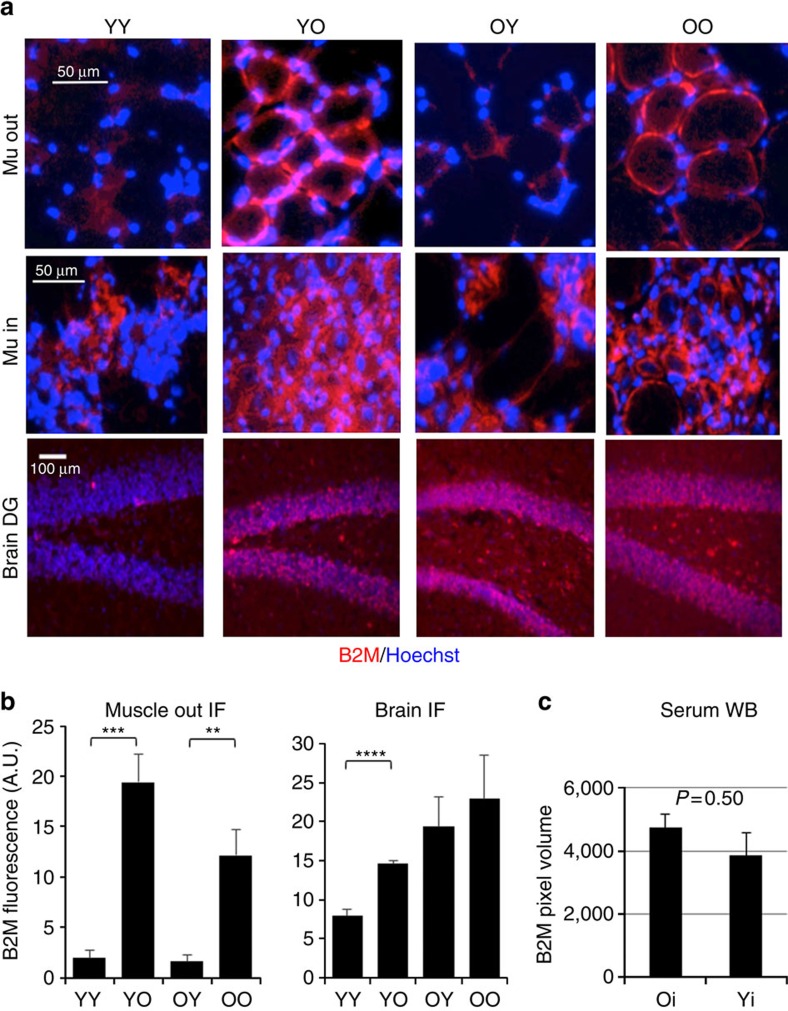
Levels of B2M in young muscle and brain correlate positively with the heterochronicity of blood exchange. (**a**) Muscle cryosections of 10 μm and 25 μm brain-SGZ cryosections of isochronically and heterochronically apheresed mice (that had experimental muscle injury) were immuno-stained for B2M and counter-stained for Hoechst to label all nuclei. Representative images were acquired at the sites of muscle injury (Mu in) outside the injury-repair (Mu out) and at the hippocampi-DG areas (brain DG), scale bar is 50 μm for muscle and liver, and 100 μm for brain. (**b**) Pixel density of B2M was quantified using Image J from serial cryosections represented in **a**; and shown are the means and standard errors. In muscle: ***,**P<0.005. Significant differences were observed between YY and YO (*P*=0.004), OY and OO (0.001), YO and OY (*P*=0.0007), and YY and OO (*P*=0.006), *N*=5–7 per group. In brain: ****P<0.00005. Significant differences were observed between YY and YO (*P*=0.00001), and YY and OO (*P*=0.004). (**c**) Western SDS–polyacrylamide gel electrophoresis was used to analyse B2M levels in one microlitre of cell-free blood serum from 5 young (Y) and 5 old (O) mice. ECL images were quantified by ImageJ and expressed as background-corrected pixel volume. *N*=5. *P*=0.5. B2M becomes increased with age in muscle and brain but it is not elevated in old blood serum as compared with young. After heterochronic blood exchange B2M is increased by old blood in young muscle and decreased by young blood in old muscle (regionally, outside of the injury site). B2M is also increased in young hippocampi-DG after exchange with old blood, but B2M is not diminished in the old DG after young blood exchange. Shown are means±s.e.m. for all histograms.

To start looking into the molecular mechanisms that are responsible for these rapid influences of circulation on tissue repair and maintenance, we studied the levels of B2M. B2M is the invariant chain of MHC class I that becomes elevated with inflammation and based on current reports is over-pronounced in old muscle and brain, as compared with young^8^,^14^,^20^. B2M levels were assayed by immunofluorescence in tissue cryosections (Fig. 4a,b) and by western blotting (Supplementary Fig. 5) in the young and old mice that underwent isochronic versus heterochronic blood exchange as described above. For tissues derived from mice injured with CTX in their TAs, the immunofluorescence on muscle and brain tissue cryosections demonstrated that exchange with old blood rapidly (within 6 days), elevated the B2M levels in young muscle located outside of the CTX injury, and in the SGZ of the young hippocampus, (Fig. 4a,b). Interestingly, the B2M remained high in the old hippocampi of the heterochronically exchanged animals (Fig. 4a,b). Furthermore, for muscle these age-specific differences in B2M were less pronounced between YY and YO cohorts and were undetectable between the OO and OY cohorts when immunofluorescence was performed at the sites of CTX injury—muscle regeneration (Supplementary Fig. 5) in agreement with the previous findings that inflammation overlaps in space and time with muscle repair and that some degree of transient inflammation is needed for successful myogenesis^7^,^21^.

Western blotting confirmed the results obtained by the immunofluorescence and demonstrated that B2M levels were increased with age in muscle and in brains, while there was no detectable age-specific increase of B2M in livers (Supplementary Fig. 5 and Supplementary Fig. 9). The regional tissue differences in B2M levels are not resolvable by the western analyses, thus the differences between the cohorts were less drastic, but in general agreement with those seen by the immunofluorescence.

The age-elevated increase of B2M was less noticeable in the muscle and brains of the animals that did not experience experimental muscle injury; for livers there was again no detectable age-specific change (Supplementary Fig. 5). By immunofluorescence, the regional (DG) age-specific difference in B2M persisted in brains of young versus old mice that did not experience muscle injury; and no significant modulation of B2M were detected between YY versus YO or OO versus OY cohorts (Supplementary Fig. 5).

The simplest explanation to these changes in tissue B2M would be an age-imposed increase in circulating B2M, which was suggested by the earlier reports^8^,^14^,^20^. Thus, we performed B2M western blotting analysis for circulating B2M in five young and five old blood serum samples. Interestingly, while there was a mild trend (∼10%) for the age-specific increase in systemic B2M, it was without statistical significance (Fig. 4c). And similar results were obtained with and without muscle injury (Supplementary Fig. 6 and Supplementary Fig. 9). Therefore, it is unlikely that systemic B2M accumulates in young tissues after heterochronic exchange, because there is no statistically significant age-imposed increase of B2M in circulation, but rapid and significant changes in B2M manifest as the result of heterochronic blood exchange and moreover, in specific regions of muscle (outside of injury) and brain (DG).

Transforming growth factor beta1 (TGF- beta1) becomes up regulated with age systemically and locally, and experimental attenuation of the age-increased TGF-beta/pSmad3 reduces B2M in muscle and brain^1^,^8^,^22^. However, while TGF-beta1 was expectedly elevated with age (myostatin and follistatin remained similar), a single procedure of heterochronic blood exchange did not significantly change the TGF-beta1/pSmad3 intensity in either young or old muscle (Supplementary Fig. 7). These data suggest that other than TGF-beta1/pSmad3 determinant(s) must account for the induction of B2M by old blood in young animals.

These results demonstrate an age-specific increase of B2M in muscle, brain, but not liver and blood serum, and establish that exchange of young mice with old blood elevates B2M in muscle and brain in a regional manner; however, no decline of B2M is observed in hippocampi or the entire brain of old mice exchanged with young blood. Moreover, the tissue increase of B2M after heterochronic blood exchange does not seem to be caused by age-elevated systemic B2M or TGF-beta1.

## Discussion

Exchange transfusion is a routine strategy for the management of several diseases, such as sickle cell disease, haemolytic disease of newborns, some cases of severe malaria and so on^23^,^24^,^25^. Plasmapheresis is currently FDA approved and used in the treatment of thrombotic thrombocytopenic purpura^26^, Guillain–Barré syndrome, Goodpasture syndrome and several other highly acute autoimmune conditions, where its main beneficial effect is to clear out offending autoimmune antibodies^27^. The apparatus and method described here enables blood exchange to be performed in the mouse and other small animals, thus allowing for well-controlled experimental tests, which can be rapidly translated to combating a number of age-related degenerative pathologies of muscle, brain and so on using human-based exchange devices that are already FDA approved.

Importantly, our work on rodent blood exchange establishes that blood age has virtually immediate effects on regeneration of all three germ layer derivatives. Unlike with parabiosis (where other organs are shared besides blood), the positive effects of young blood benefit old muscle regeneration, while negative effects of old blood dominate in young brain (hippocampus) with liver being intermediate. The positive effects of young blood could also be explained by dilution of old blood, and not necessarily by young factors. These positive effects manifested on muscle and liver even when no additional exchanges were performed, while old animals kept producing their ageing factors for 6 days after the blood exchange with young mice. For muscle, when tested at 5 days after injury, formation of new myofibers signifies productive responses of the muscle stem cells (satellite cells) at 24 h after the injury (or earlier); hence the effects of blood exchange must have been rapid. In future work it will be interesting to establish if the long-term muscle tissue health (innervation, vascularization and so on) and muscle physiology also improve in old mice transfused with young blood, as here we focused on the acute regenerative outcome, for example, formation of the new muscle fibres at the sites of experimental injury. However, in support of currently used approach, the acute 5 days post injury regenerative outcome has been shown to reliably reflect the regulation, aging and rejuvenation of myogenesis by parabiosis and by defined factors, which moreover were found to be conserved between mice and human^1^,^2^,^3^,^4^,^5^,^6^,^7^,^8^.

Comparing neurogenesis in the absence and presence of experimental muscle injury, we uncovered that neurogenesis declines more severely in young mice exchanged with old blood when peripheral tissue is injured. This could be due to many mechanisms (inflammation being one), and our B2M data supports that B2M is up-regulated during inflammation. Importantly, these comparative studies have also demonstrated that even in the absence of muscle injury there is no enhancement of old neurogenesis by one exchange of young blood, and there is still a decline in young neurogenesis after old blood exchange.

It is also interesting that a single procedure of heterochronic blood exchange resulted in a strong inhibition of young neurogenesis, even when young systemic and local factors were produced for the remaining 6 days and the young mouse had a chance to neutralize or remove the inhibitors of the old systemic milieu. These effects reveal a dominant, rapid and lasting age-specific influence of the systemic milieu on the regenerative capacity of multiple tissues.

While these results are seemingly different from those obtained by young plasma infusions into old mice, as mentioned above, neurogenesis has not been tested in those previous studies^14^, only cognitive performance which may be independent of neurogenesis. Additionally, it remains possible that with repeated infusions of young plasma some positive effects would manifest for more parameters of the aged brain. When interpreting the results of functional performance (four-limb hanging test) it is more likely that lack of performance of the young mice after blood exchange with old blood stems from a cognitive decline in addition to diminished muscle strength and endurance. Young and old mice had initially similar hanging indices in the training session, and while the YY cohort significantly enhanced its performance after training, the old mice and the young mice transfused with old blood did not. Additionally, it is unlikely that muscle strength declined dramatically in just 6 days without a detectable decrease in muscle tissue health or regeneration (for example, by histology). The four limb hang test requires coordination, endurance and strength, which might diminish in mice by 2 years of age; however, our old mice were robust and active (as per daily observations of our scientists and University vets), thus a complete lack of improvement in the four limb handing test is not because animals were too frail physically. It is hence surprising, given the reported improvement in Morris water maze after infusions of young plasma (where not only learning but strength and coordination and also vision are of importance)^11^,^14^.

As mentioned, repeated infusions might have cumulative effect, however, the volume of young systemic factors is ∼50% of the total after blood exchange, as compared with 5–10% after each plasma injection^11^, and old circulatory factors are removed by exchange, but not in a plasma injection protocol. Of note, there is a number of different tests for animal learning and cognition and some of them, but not others might reveal the improvement of old mice injected with young plasma or transfused with young blood; this direction would be interesting to pursue in future work.

We did not perform additional heterochronic parabiosis studies in parallel with the heterochronic blood apheresis. There might be slight differences in the regeneration indices and so on phenotypes, not only between the individual experiments, but also even between different pairs of animals in individual heterochronic parabiotic pairings. However, as demonstrated by others and us repeatedly over the past decade^1^,^2^,^3^,^4^,^5^,^6^,^7^,^8^, the enhancement of old tissue regeneration and inhibition of young tissue regeneration seen in heterochronic parabiosis has been robustly reproducible validating a comparison of our new data.

Identification of the blood factor(s) that rapidly influence the health of several tissues that we report here, and uncovering their mechanism of action would certainly be a worthy future endeavour. In this regard, the rapid, significant and tissue regional increase in B2M on transfusion of young mice with old blood could contribute to the cognitive, as well as neurogenic and myogenic changes^8^,^14^,^20^. It is interesting that regeneration of young muscle does not diminish after a transfusion of old blood and the levels of B2M differ between the non-injured and injured-regenerating regions of muscle; muscle regeneration overlaps in space and time with inflammation and some inflammatory cytokines are positive regulators of myogenesis^7^,^28^,^29^. This might explain the higher tolerance of injured muscle to old blood, as compared with the brain-neurogenesis and diminished capacity to improve hanging test performance after the training session. Similarly, while muscle injury seems to enhance hepatogenesis, such an increase might be physiologically important in young mice and its lack in old animals might reflect a negative age-related decline that is quickly restored by the young blood. The lack of age-specific changes in B2M in livers is also interesting and further promotes the idea that for tissues with high rate of regeneration, some inflammation might be a positive factor.

Some of the broadly functioning inhibitory culprits of the aged systemic and local niches of stem cells have been defined, for example, TGF-beta1, osteopontin and the secretome of senescent cells^7^,^8^,^30^,^31^. While it is unlikely that TGF-beta1 crosses the blood–brain barrier (BBB) and heterochronic blood transfusion does not significantly change TGF-beta 1 levels in muscle, excessively high TGF-beta1 (as found locally in tissues and in circulation in old mice and humans) can promote vascular changes leading to the leakiness of the BBB, as well as inhibit neurogenesis indirectly via inflammation and contribute to broad (other than brain) tissue pathologies^1^,^2^,^3^,^4^,^5^,^6^,^7^,^8^. A positive factor that diminishes with age systemically and is needed for muscle repair and brain health has been identified: oxytocin—a peptide hormone. Systemic administration of which enhances old muscle repair, osteogenesis and combats obesity^6^,^32^,^33^,^34^,^35^. Fibroblast growth factor (FGFs) that are secreted by human embryonic stem cells have been also shown to have positive effects on muscle and neural precursor cells and some FGFs are endocrine^3^. It will certainly be interesting to have a closer look at the TGF-beta/pSmad, bone morphogenic protein (BMP), oxytocin/MAPK, FGF/MAPK, Delta/Notch and so on cell-fate regulatory age-specific pathways in the settings of heterochronic exchange in future work. Since whole blood and not just plasma were used in our system, it would be also interesting to separate humeral and cellular influences of heterochronic blood exchange in future embodiments of the device. Of note, little has been reported of heterochronic bone marrow transplant effects on multi-tissue regeneration, and that experimental system is quite different from heterochronic blood exchange, as bone marrow transplants require lethal whole-body irradiation followed by long-term recovery of hosts.

In our experiments, it is not likely that old leucocytes crossed the young blood–brain barrier to inhibit neurogenesis in just 6 days, but positive effects of young leucocytes in wound healing and negative peripheral effects of the aged leucocytes could play a role. There was no time to develop persistent inflammation and no signs of acute inflammation were observed in our experiments, when analysing young muscle tissue histology by H&E after transfusion with young or old blood. However, in old muscle inflammation is typical (without and with transfusion); and inflammation could also differ between different organs of the same animal. In future studies, we plan to separate the effects of plasma from those of leucocytes (by adding cell-removal module to our device). Finally, from theoretical and clinical perspectives it will be interesting to comprehensively delineate the onset and duration of the positive and negative influences of the heterochronic systemic milieu on regeneration of studied here and other tissues.

Small animal blood exchange and parabiosis are different in many ways, including the timing of blood exchange, involvement of the immune system (that is, parabiotic disease), participation of the organ systems, environmental enrichment and pheromones and so on; thus, it is not surprising that some of the effects on tissue regeneration turned out to be different. However, interestingly many of the effects are quite similar and take place rapidly after the heterochronic blood exchange procedure. Summarily, current work establishes a paradigm of the plasticity of age; the age of tissues is acutely, rapidly, effectively reversed to younger or older states, and unlike parabiosis, extracorporeal blood manipulation provides a modality of rapid translation to human clinical intervention.

## Methods

### Animals

All procedures were performed in accordance with the administrative panel of the Office of Laboratory Animal Care, UC Berkeley and the protocol was approved by the UC Berkeley Animal Care and Use Committee. Young male C57BL/6 mice (2 months of age) were purchased from the Jackson Laboratory (#00664). Twenty-two-month-old male C57BL/6 mice were purchased from the National Institute on Ageing.

### Blood exchange

Blood exchange in young animals was performed at 3 months of age and in old animals at 23 months of age. The procedure was as follows: first a jugular venous catheter was inserted in the right jugular vein as we did previously using refinements by Bardelmeijer *et al*.^16^,^36^. Using a 10 μl Hamilton syringe 10 μl of catheter locking solution containing 500 units per ml lithium heparin in 90% glycerol and 10% phosphate-buffered saline (PBS) were introduced into the catheter to prevent clot formation. The animals were allowed to heal from the surgical procedure for 24 h and then they were immobilized with isoflurane anaesthesia at a 1% concentration. The locking solution was removed from their catheters and a bolus of PBS containing 0.5 units per μl lithium heparin was administered IV at 100 units per kg and they were connected to our blood exchange apparatus. Briefly, using our microfluidic blood exchange device 150 μl of blood was transferred from one mouse to another 15 times with a 30 s delay between blood administration and withdrawal twice within 24 h to yield a <90% homogenization of blood between the two animals. We visualized the blood coming from one animal and going into another (Supplementary Movie 1) and we calculate that after a single exchange session the two animals blood is ∼90% homogenized, and are able to programme precisely the degree of homogenization we desire and confirm the volumes of exchanged blood in each experiment (knowing the tubing diameter and length). The mice are weighed before the exchanges and we calculate the volumes of exchanged blood based on fluid dynamics as well as the outcome of many control experiments in which total blood volume is correlated with animal weight.

### Cardiotoxin muscle injury

Eight hours after the blood exchange procedure, mice were injured by intramuscular injections of CTX (Sigma, 10 μl per muscle at 0.1 μg ml^−1^) into the tibialis anterior (TA). Five days after the injury, TA muscles were isolated.

### Tissue isolation

Tissue isolation was performed postmortem and muscle, liver and brain were snap frozen in isopentane (−70 °C), embedded frozen in tissue-tek optimal cutting temperature (OCT, Sakura Finetek, The Netherlands).

### Tissue sectioning

OCT embedded muscle and liver were sectioned using a cryostat at 10 μm. Sagittal sections of brain hippocampi were similarly processed to 25 μ sections. The respective tissue sections were then attached to positively charged glass coverslip slides in preparation of H&E or immunohistochemistry analysis.

### H&E staining

Histological preparation for muscle sections was performed: dehydration and removal of OCT in 70% ethanol for 3 min, dehydrated at 95% ethanol, hydrated in deionized water for 1 min, haematoxylin for 5 min, 1X Scott's water for 1 min, rinsed in water for 1 min, eosin for 4 min, rinsed in water for 30 s, a dehydration series of 70%, 95% twice, 99 and 100% ethanol, for 1 min each. Sections were cleared with xylenes twice, 1 min each. 2–3 drops of 50% resin/50% xylenes mounting medium were added to each slide and glass cover slips were placed. Images of injury sites were obtained by light microscopy.

### Immunofluorescence

Primary antibodies were used at 0.5–1 μg ml^−1^ and sourced from Abcam: rabbit anti-Ki67 (ab16667, 1:200), and rabbit anti-pSmad3 (1:200); Fisher Scientific: rabbit anti-follistatin (PA519787, 1:200); Santa Cruz Biotechnologies: anti-Sox2 (sc-17320 1:400); R&D Systems mouse anti-Albumin (MAB1455, 1:1000), rabbit anti-myostatin (AF788, 1:200). Mouse hybridoma anti-eMHC was prepared in house (F1.652 clone, Developmental Studies Hybridoma Bank, University of Iowa, deposited by Blau, HM) and used at 1:50., Secondary fluorochrome conjugated antibodies were from Life Technologies: goat anti-rabbit 546 (A11010, 1:2,000) and goat anti-mouse 488 (A11029, 1:2,000). DNA was stained by Hoechst 33342 from Sigma Aldrich (B2261) used at 1 μg ml^−1^. Negative controls with isotype-matched immunoglobulin G (IgGs) were routinely performed for all immunodetection studies shown here and above; and background non-specific immunofluorescence was minimal, Supplementary Fig. 8.

### Sections

Sections were prepared using the following methods: sections were blocked in 1% staining buffer (1% calf serum in 1X phosphate saline buffer, PBS) for 30 min on positively charged frosted glass microscope slides. Sections were then incubated with primary antibodies overnight at 4 °C, washed three times in staining buffer and secondary antibodies and Hoechst nuclear DNA were added for 2 h at room temperature, followed by 3 PBS washes, and mounting with Fluoromount (Sigma F4680). Samples were imaged with a Zeiss Axioscope fluorescent microscope. Samples stained for embryonic myosin heavy chain were not fixed nor were they permeablized. For B2M, myostatin and follistatin, muscle sections were fixed in 70% ethanol for 8–12 h at 4 °C and washed with 1 × PBS, permeabilized with 0.1% Triton X-100 for 5 min at room temperature before adding antibodies as above. TGF beta1 and pSmad 3 were stained with a protocol identical to those of myostatin, follistatin, and B2M, only without any fixation. Brain sections were fixed with 4% paraformaldehyde for 5 min. Sections were permeablized by 0.1% Triton X-100. Liver sections were fixed with 70% ethanol, permeablized by 0.1% Triton X-100.

### Oil red staining

. Liver sections of 10 μ were hydrated in 1 × PBS for ∼10 min. The sections were then washed in 60% isopropanol for 3–5 min and later placed in isopropanol-based Oil Red staining solution for 15 min. After, the sections were washed in 60% isopropanol once more for 1 min. Nuclei on these sections were stained after a 5-min wash in haematoxylin. The sections were finally washed in deionized water for 1 min. Fluoromount was used as the mounting medium and images were taken from these slides.

### Western blotting

Tissue lysates were prepared using T-per tissue protein extraction buffer (Thermo Fisher prod #78510) according to manufacture's protocol. Protein quantification was done using RED 660 protein assay (Cat #786–676). Lysates and blood were diluted to 1 × Laemmli buffer, boiled for 5 min and 30 μg protein or 1 μl blood was separated on precast 4–20% TGX gels (Bio-Rad) and transferred to 0.2 μm polyvinylidene difluoride membranes (Millipore). Primary antibodies (B2M, Abcam, ab75853, 1:5,000, gapdh, Abcam, ab9485, 1:3,000, and actin, Fisher scientific, MA5–15739, 1:3,000, anti-TGF-beta-1 from R&D Systems, MAB240, 1:1,000) were diluted in PBS+0.1% Tween-20, 5% non-fat dry milk, incubated with overnight at 4 °C. horseradish peroxidase (HRP)-conjugated secondary antibodies (goat anti-rabbit IgG-HRP, Santa Cruz Biotechnology, sc-2004, 1:1,000, goat anti-mouse IgG-HRP, Santa Cruz Biotechnology, sc-2055, 1:1,000) were diluted in PBS+0.1% Tween-20, 1% BSA and incubated for 1 h at room temperature. Blots were developed using Western Bright ECL reagent (Avansta), and analysed with a Bio-Rad Gel Doc/Chemi Doc Imaging System and Quantity One software. Results of multiple assays were quantified using Image J software. Pixel Intensity of bands of interest were normalized with pixel intensity of glyceraldehydes-3-phosphate dehydrogenase or β-actin. Uncropped images of immunoblots are shown in Supplementary Figs 9 and 10.

### Four limb hanging test

Muscle strength and endurance were determined by timing how long mice can hang upside down from a wire screen, as published^19^. Briefly, each mouse was placed on a 1 cm. mesh, 1 mm wire screen, 30 cm over soft bedding, inverted and timed until the mouse fell. Each animal was given two to three trials with at least a 5 min rest between trials. Hanging index is expressed as the maximum hang time times the weight of the mouse, with longer times or greater weight for a given time considered better performance.

### Data quantification

Muscle regeneration indices were calculated by counting percent de-novo myofibers with central nuclei to total nuclei in 3–4 representative images of 10-μ muscle sections for each blood exchange cohort. Muscle fibrosis was quantified by measuring fibrotic areas in arbitrary units on Image J. These fibrotic areas were normalized to the area of the image taken at × 20 (∼1,4000 μm^2^). Neurogenesis was quantified by counting the number of Ki67+/Hoechst+ cells in 175 micrometre (from multiple 25 micrometre sections) of the SGZ each blood exchange cohort and extrapolated to the total thickness of the DG. Sox2+/Ki67+ cells were quantified by counting the number of these cells per 25 μm section, one section per noted cohort. Counting cell numbers from multiple 10-μm sections of each blood exchange cohort did quantification of hepatocyte proliferation, for example, Ki67+, albumin+, and Hoechst+ cells and of albumin- Ki67+ fibrotic cells. Oil Red quantification was done by obtaining the total area of the red fatty droplets as a percentage of the entire × 20 image area used, using a consistent color-threshold function in software ImageJ. B2M in muscle, liver and brain was quantified by measuring areas of fluorescence in the images on Image J (identical areas were applied to each image). Measured in arbitrary units, the area for muscle spanned the entire image, the area measured in brain was ∼2 in the DG, and the area measured in liver was 8. The fluorescence densities were corrected by subtracting respective background IgG fluorescence.

### Statistics

A non-paired, two-tailed *t*-test in Microsoft Excel was performed on all of the respective data. *P* values of 0.05 or less were considered statistically significant.

### Data availability

The data that support the findings of this study are available from the corresponding author upon request.

## Additional information

**How to cite this article:** Rebo, J. *et al*. A single heterochronic blood exchange reveals rapid inhibition of multiple tissues by old blood. *Nat. Commun.* **7,** 13363 doi: 10.1038/ncomms13363 (2016).

**Publisher's note:** Springer Nature remains neutral with regard to jurisdictional claims in published maps and institutional affiliations.

## Supplementary Material

Supplementary Information Supplementary Figures 1-10

Supplementary Movie 1 This movie shows a part of the blood exchange procedure.
