# Supplementary material for: A single heterochronic blood exchange reveals rapid inhibition of multiple tissues by old blood
Source: Nat Commun. 2016 Nov 22;7:13363. doi: 10.1038/ncomms13363 (PMC5121415; doi:10.1038/ncomms13363)
Supplement: Supplementary Information — Supplementary Figures 1-10 [file ncomms13363-s1.pdf]

Supplementary Figure 1.

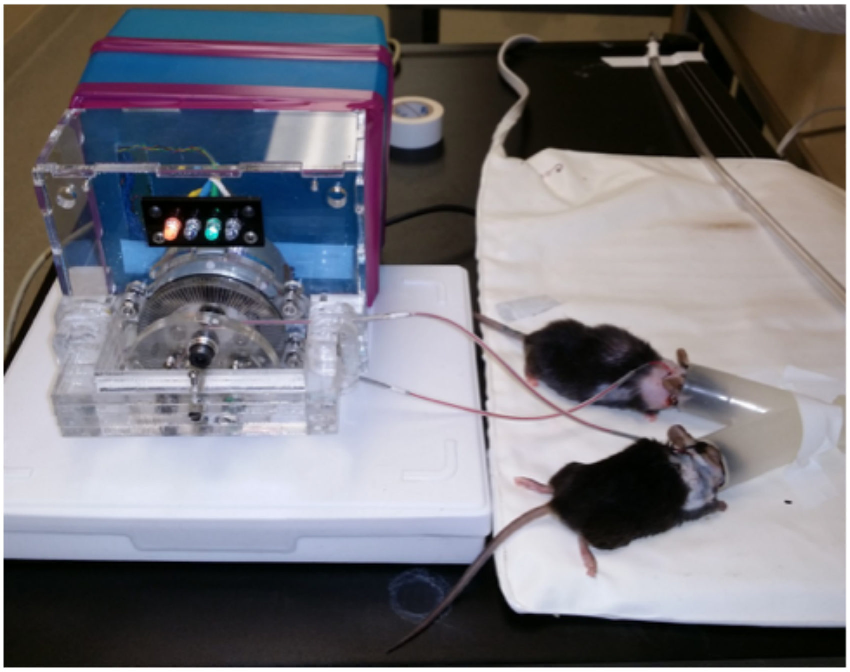

Supplementary Figure 1: Small animal blood exchange device.
The device is approximately 10 cm by 10 cm by 15 cm. LED lights provide status. The device is fully programmable and computer controlled. The peristaltic pump, like the rest of the device, communicates with a host controller using a USB port. The commands that can be issued to the device cover modes of stepping the motor, speed and direction of each individual step, changing the color of each individual LED in the status area. The device operates similarly to (*16*).

Supplementary Figure 2.

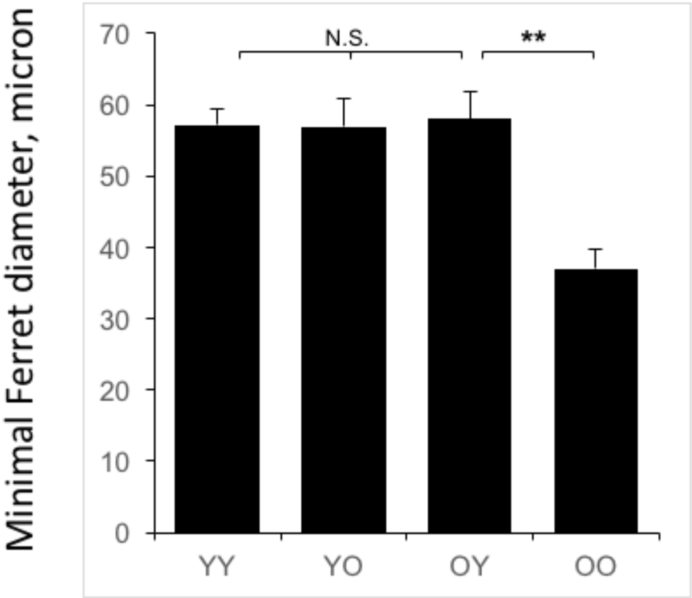

Supplementary Figure 2: Minimal Feret diameter of newly formed old myofibers is enhanced by young blood apheresis.

Minimal Feret diameter of newly-formed myofibers with central nuclei was measured as published (*6, 18*) using H&E staining and was found to decline with age (consistently with previous reports (*6*) and to be increased in muscle of old mice after one heterochronic blood apheresis. No decline in minimal Feret diamenter was observed in newly formed myofibers of young mice apheresed with old blood. N=4 independent blood exchange experiments, p=0.005.

Supplementary Figure 3.

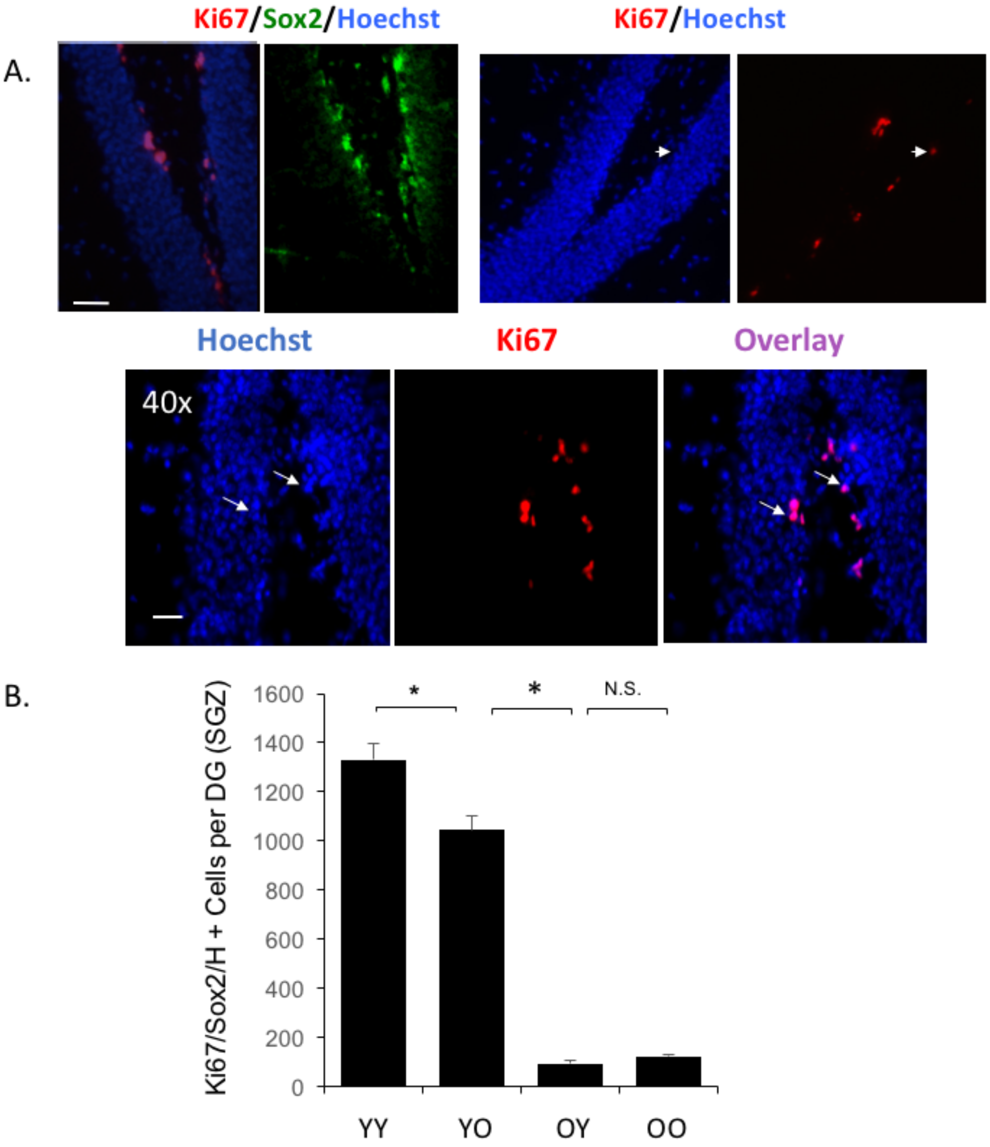

Supplementary Figure 3. SGZ neurogenesis in YY, YO, OY and OO mice that did not have muscle injury.

A. Representative image of YY Ki67+/Sox2+ SGZ cells in animals that did not receive muscle injury and representative images of Ki69/Hoechst. Virtually all Ki67+ve nuclei are Sox2+ve and Ki67 signal is nuclear. Scale bar=100micron.

B. Quantification of the Ki67+/Sox2+/Hoechst+ cells per SGZ (using serial 25 micron cryosections) of the YY, YO, OY and OO mice that did not have experimental muscle injury. The number of Ki67+/Sox2+ cells in YO animals has also significantly decreased when compared to YY animals (p=0.03), as seen in the neurogenesis trend with animals that have received experimental muscle injury. *=p=0.05, N=3 independent blood exchange experiments per each age. The numbers of YY cohort were set to 100% in Figure 2.

Supplementary Figure 4.

A.

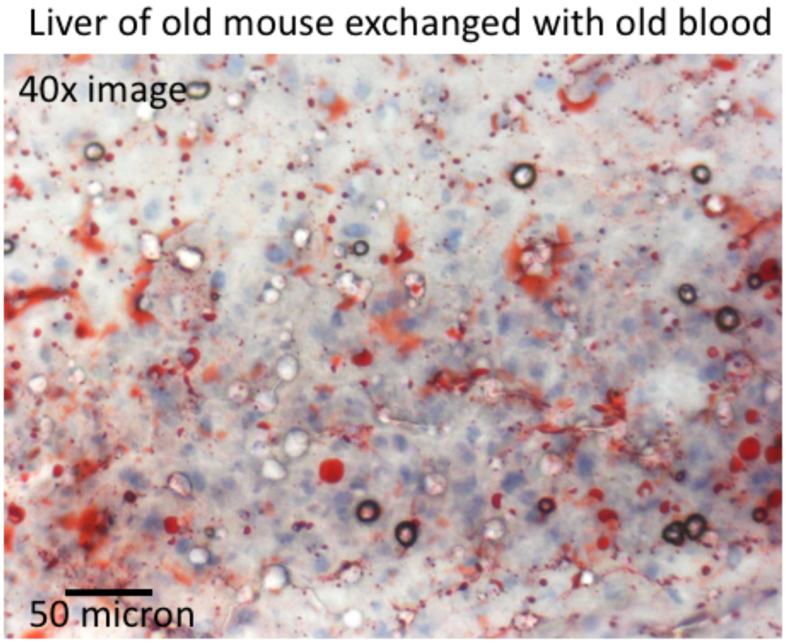

B.

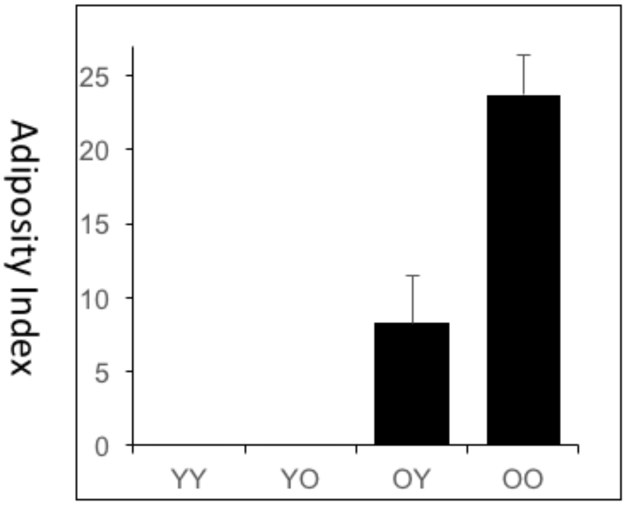

Supplementary Figure 4. Young liver adiposity does not change by the heterochronic blood exchange.
Oil Red assay was performed on 10 micron liver cryosections, as described above in Figure 4 and in Methods.
A. 40x representative image of OO liver is shown to illustrate significant liver adiposity.
B. While exchange with young blood significantly reduces old liver adiposity (N=3, P>0.05), young isochronic and young-old heterochronically exchanged mice have negligible adiposity and no statistically significant difference between YY and YO cohorts is detected. N= 3-5, p< .05 = *, p < .005 = **

Supplementary Figure 5.

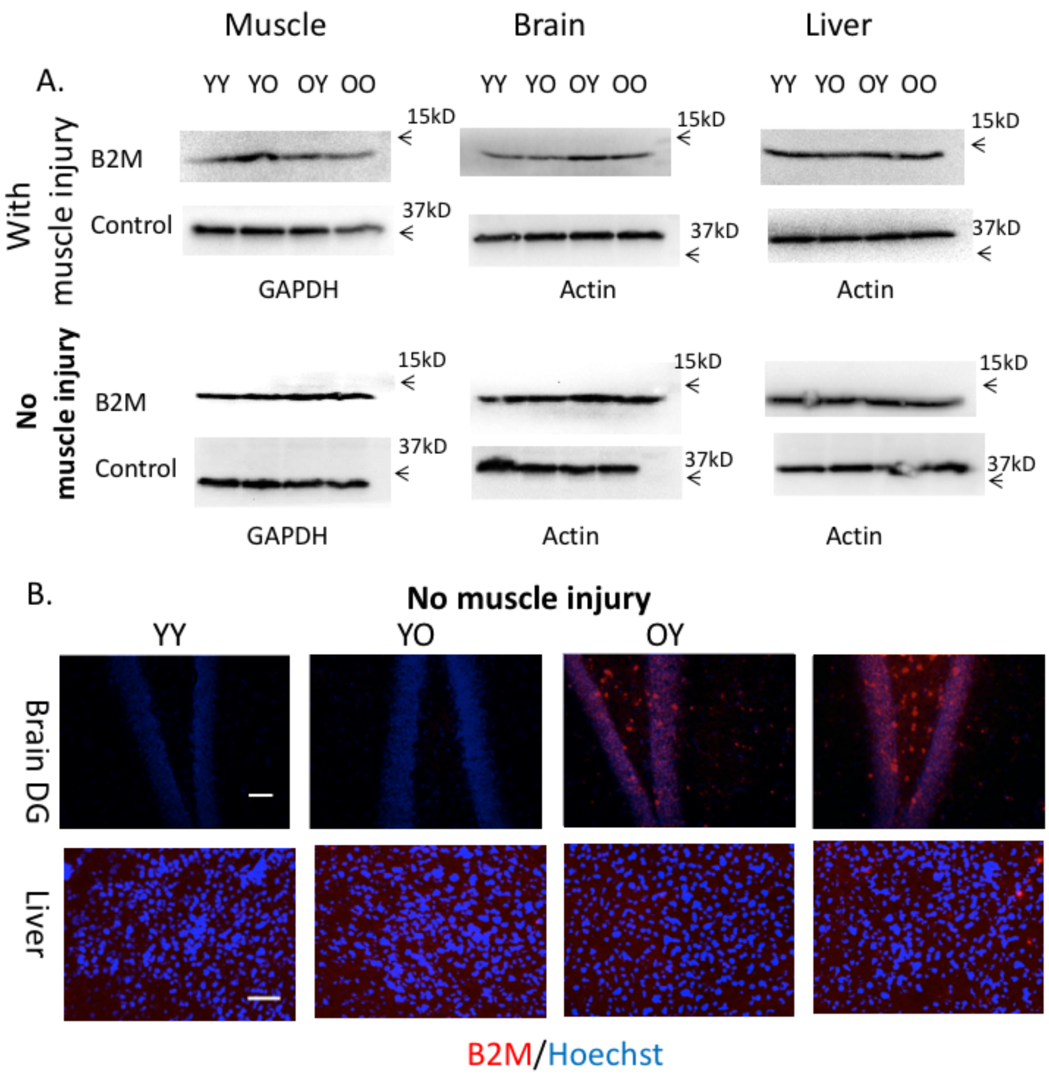

Supplementary Figure 5: B2M levels in tissues of injured and non-injured mice.
A. Western Blotting with B2M specific antibodies and loading controls: GAPDH and actin antibodies was performed using lysates of muscle, brain and liver derived from isochronically and heterochornically apheresed mice that had experimental muscle injury as well as mice that had no muscle injury. Age-specific elevation of B2M was detected in muscle and in brain, but not in liver; muscle injury resulted in more pronounced difference between the young (low) and old (high) B2M levels in muscle and brain.
B. Immunofluorescent images were acquired at hippocampi-DG and livers of isochronically and heterochornically apheresed mice that did not experience muscle injury; and from the sites of acute muscle injury-regeneration. While the age-specific elevation of B2M persists at DG regions of these mice, the B2M immunodetection is similarly low at DG of young mice transfused with either young or old blood. No differences in B2M levels between the studied cohorts were detected in livers by the immunofluorescence. B2M levels are high at the sites of acute muscle injury with minimal-no difference between the studied cohorts.

Supplementary Figure 6.

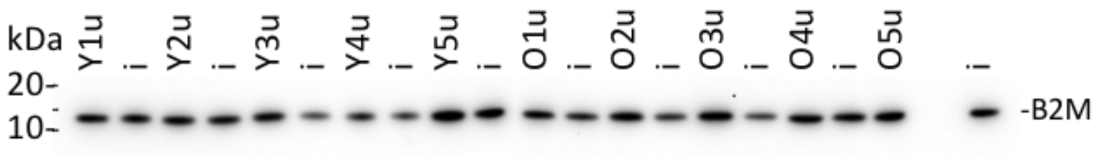

Supplementary Figure 6. Western Blotting detection of B2M in cell-free blood serum of 5 young and 5 old mice (cohorts with and without muscle injury).

1 ul of cell free blood serum from 5 young and 5 old mice (injured in TA and Gastroc by CTX or uninjured) was resolved on SDS-Page Western Blot. While there were some differences between individual mice, and a general increase in B2M with injury, no significant differences were detected between young injured (Y#i) to old injured (O#i) and young uninjured (Y#u) to old uninjured (O#u) samples.

Supplementary Figure 7.

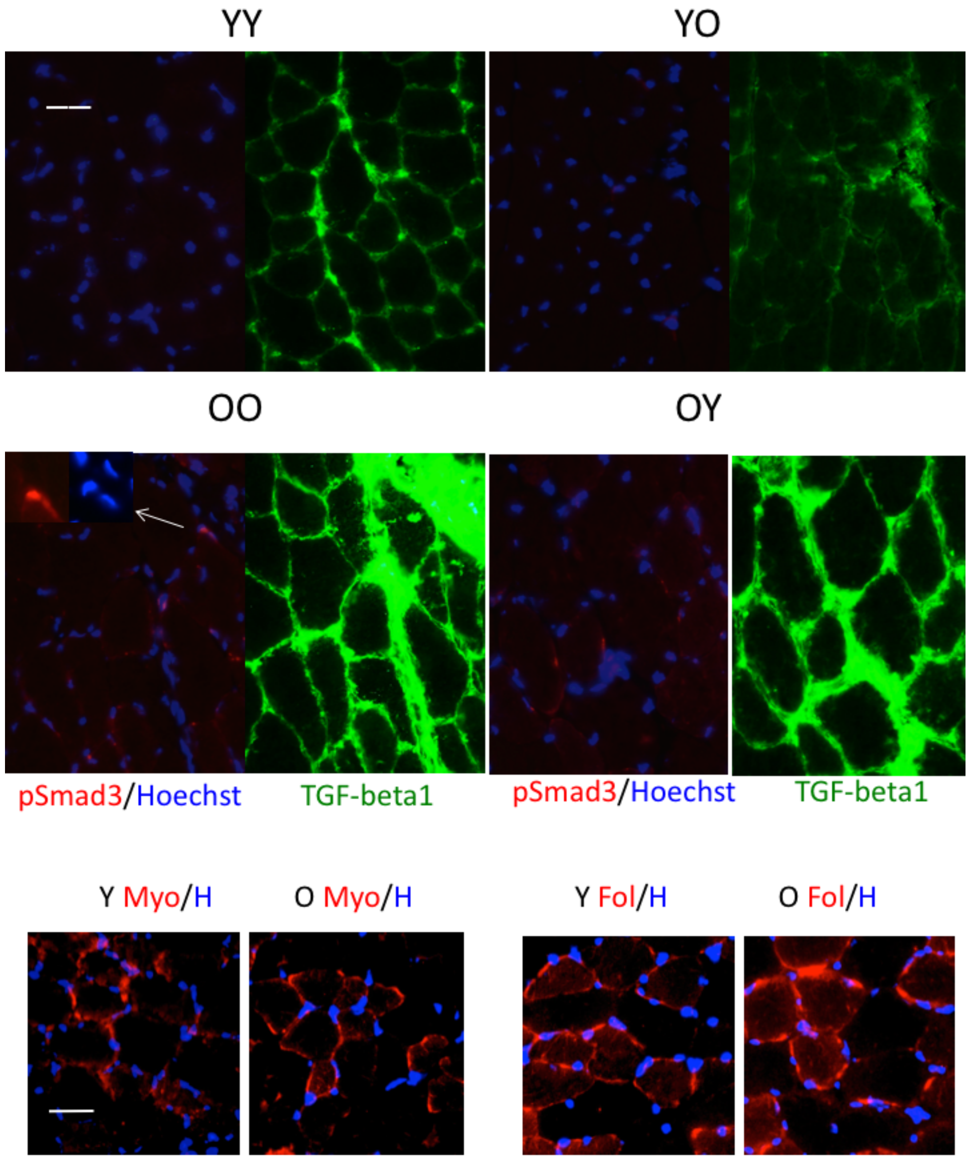

Supplementary Figure 7. TGF-beta1, pSmad3, Myostatin and Follistatin immunofluorescence in muscle.

Co-immunodetection for TGF-beta1 (green) and pSmad3 (red) was performed in 10 micron uninjured TA muscle sections derived from YY, YO, OY and OO mice. Hoechst (blue) was used to label all nuclei. Myostatin and Follistatin (both red) were also immunodetected in TA muscle cryosections from YY and OO mice, using Hoechst (blue) to label nuclei. While age-specific increase in TGF-beta1 and pSmad3 is pronounced as previously reported (*1, 8, 22*), heterochronic blood transfusion does not change the levels of these proteins in either young or old muscle. A larger image of pSmad3+ cells is shown (white arrow) to illustrate the nuclear localization. For myostatin (Myo) and Follistatin (Fol) the levels varied within each muscle, but no significant differences were detected between young and old mice, in agreement with our previous report (*22*), Hoescht nucle in blue. All scale bars= 50 micron.

Supplementary Figure 8.

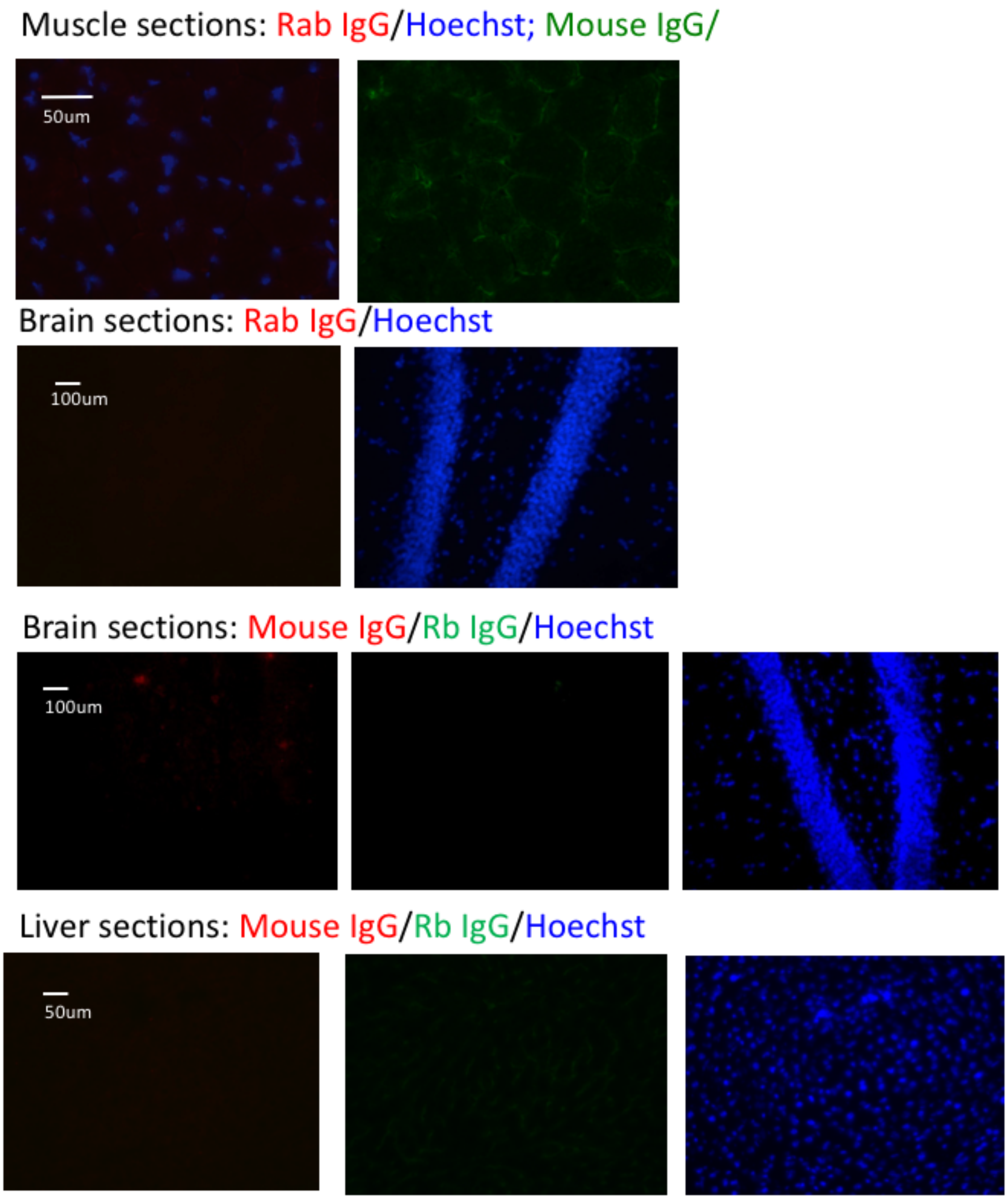

Supplementary Figure 8. Representative negative controls for immunofluorescence. Isotype-matched IgG controls were routinely performed for all antibody combinations and shown are representative images for such IgG controls in muscle, brain and liver tissue sections. Using protocols described in Methods produces very low non-specific antibody fluorescence. Scale bars for muscle and liver are 50 micron and 100 micron for brain.

Supplementary Figure 9.

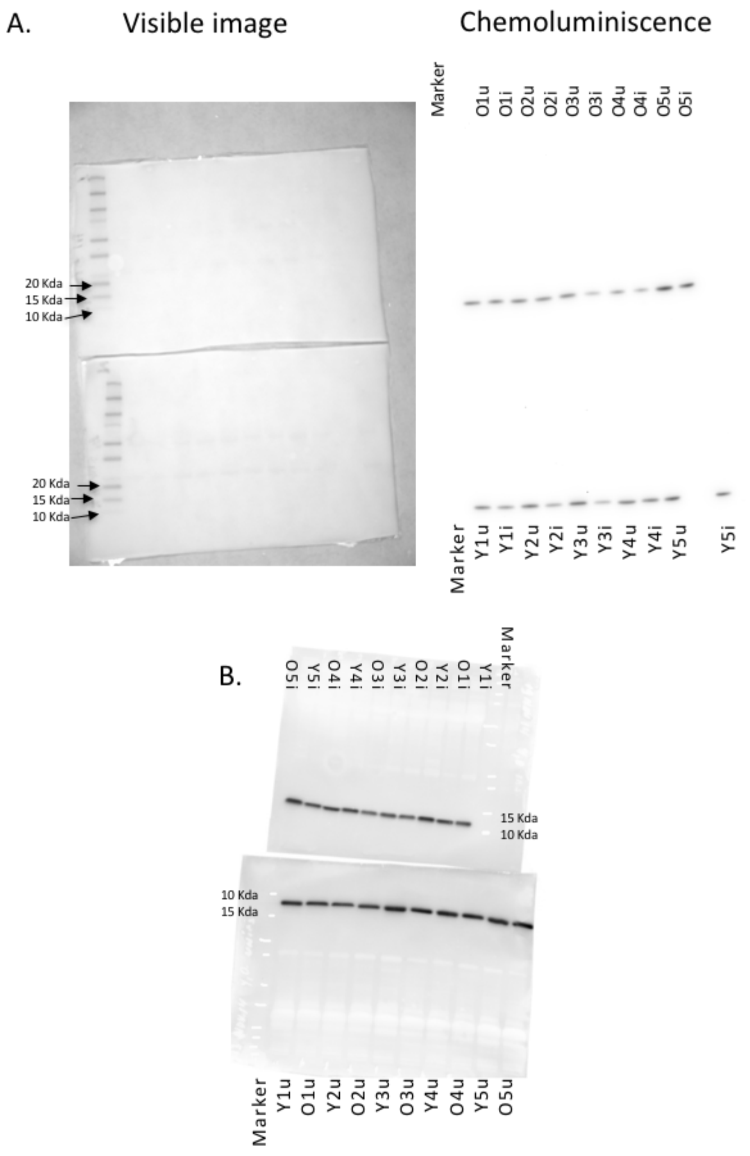

Supplementary Figure 9. Primary data for Figure 4C and Supplementary Figure 6.
A. Visible image with visible Molecular Weight (MW) marker, and Chemoluminiscence image are photographed for the same Western Blot and MWs (20-10kDa) are shown; the single B2M band of predicted MW (12kDa) is seen by Chemoluminiscence.
B. A separate Western where the same young and old blood samples are loaded: the injured are on top blot and uninjured on the bottom blot. The Visible image was temporarily overlayed to the Chemiluminescent image and the MW markers were marked, so they are visible on that blot. Once again the single B2M band of predicted MW is seen.
There is no difference between the 5 young and 5 old serum samples in the B2M levels in either A or B.

Supplementary Figure 10.

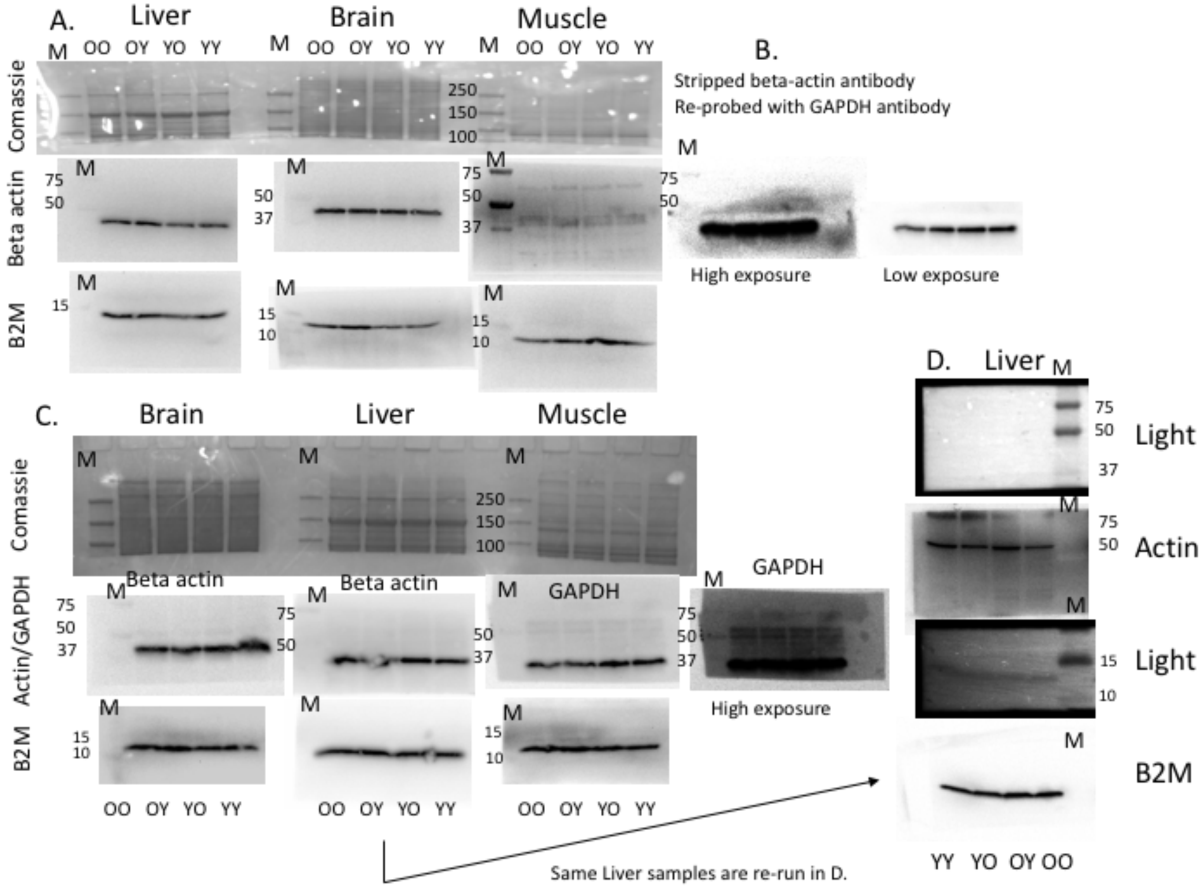

Supplementary Figure 10. Primary data for Supplementary Figure 5A and B.
A Shows samples from animals that had muscle injury (Supplementary Figure 5A), and **C** shows samples from mice that did not have muscle injury (Supplementary Figure 5B). M indicates molecular weight marker bands for all blots. The tops of the Western gels above 100kDa were cut and used for Commassie staining to confirm equal sample loading, and the bottoms were used for transfer to membranes, followed by probing with the indicated antibodies. The blots were cut into strips: 20-75kDa to probe with the loading control antibodies, and below 20kDa strips were probed with B2M antibodies. **B.** The muscle samples blot (from A) that was probed with anti-actin antibody showed multiple bands and was stripped and re-probed with anti-GAPDH antibody as a different loading control, which worked well to show one specific band (dark and light exposures are shown to illustrate the molecular weight marker bands on the dark exposure background). A very dark exposure that shows some visible molecular weight markers as background bands is also shown for GAPDH in **C**.
D. Since the liver samples in the B2M Western with no muscle injury had too low background to see the molecular weight marker bands, the same samples were re-run on another Western Blot with visible light exposures taken to show the markers, followed by the Chemoluminiscence imaging of the same blot. A single protein band after immunodetection with B2M specific antibodies is seen in all strips that are cut at 20kDa.
